# B Cell Lymphocytes as a Potential Source of Breast Carcinoma Marker Candidates

**DOI:** 10.3390/ijms25137351

**Published:** 2024-07-04

**Authors:** Soňa Tkáčiková, Miroslav Marcin, Peter Bober, Mária Kacírová, Michaela Šuliková, Jozef Parnica, Dávid Tóth, Marek Lenárt, Jozef Radoňak, Peter Urdzík, Ján Fedačko, Ján Sabo

**Affiliations:** 1 Department of Medical and Clinical Biophysics, Faculty of Medicine, University of Pavol Jozef Šafárik in Košice, Trieda SNP 1, 04011 Košice, Slovakia; miroslav.marcin@upjs.sk (M.M.); peter.bober@upjs.sk (P.B.); michaela.sulikova@upjs.sk (M.Š.); jozef.parnica@upjs.sk (J.P.); 2 Center of Clinical and Preclinical Research MEDIPARK, Faculty of Medicine, University of Pavol Jozef Šafárik in Košice, Trieda SNP 1, 04011 Košice, Slovakia; maria.kacirova@upjs.sk (M.K.); jan.fedacko@upjs.sk (J.F.); 3 Department of Gynaecology and Obstetrics, Faculty of Medicine, University of Pavol Jozef Šafárik and UNLP in Košice, Trieda SNP 1, 04011 Košice, Slovakia; david.toth@upjs.sk (D.T.); peter.urdzik@upjs.sk (P.U.); 4 1st Department of Surgery, Faculty of Medicine, University of Pavol Jozef Šafárik and UNLP in Košice, Trieda SNP 1, 04011 Košice, Slovakia; marek.lenart@student.upjs.sk (M.L.); jozef.radonak@upjs.sk (J.R.)

**Keywords:** peripheral blood, lymphocyte B, carcinoma, proteomic analysis, mass spectrometry, CD19+ marker

## Abstract

Despite advances in the genomic classification of breast cancer, current clinical tests and treatment decisions are commonly based on protein-level information. Nowadays breast cancer clinical treatment selection is based on the immunohistochemical (IHC) determination of four protein biomarkers: Estrogen Receptor 1 (ESR1), Progesterone Receptor (PGR), Human Epidermal Growth Factor Receptor 2 (HER2), and proliferation marker Ki-67. The prognostic correlation of tumor-infiltrating T cells has been widely studied in breast cancer, but tumor-infiltrating B cells have not received so much attention. We aimed to find a correlation between immunohistochemical results and a proteomic approach in measuring the expression of proteins isolated from B-cell lymphocytes in peripheral blood samples. Shotgun proteomic analysis was chosen for its key advantage over other proteomic methods, which is its comprehensive and untargeted approach to analyzing proteins. This approach facilitates better characterization of disease-associated changes at the protein level. We identified 18 proteins in B cell lymphocytes with a significant fold change of more than 2, which have promising potential to serve as breast cancer biomarkers in the future.

## 1. Introduction

Breast cancer (BC) is the most common cancer in women, with 2.3 million new cases diagnosed per year, leading to 690,000 deaths annually [1]. To date, disease prognosis and treatment stratification are heavily reliant on tumor, nodal, and metastasis staging and breast cancer subtypes. Based on IHC staining for ESR, PGR, and HER2, the majority of breast cancer cases are classified as ER positive (~65%), followed by HER2 positive (~20%) and triple-negative breast cancers (TNBCs; ~15%) [2,3,4]. With the combination of the presence of positivity for these receptors, breast tumors fall primarily into three major classes, i.e., luminal (A and B subtype), HER2 overexpression, and triple-negative phenotypic tumors (TNPs), with triple-negative tumors being the most heterogeneous and comprising largely the basal subtype. Luminal-like tumors express hormone receptors, with expression profiles reminiscent of the luminal epithelial component of the breast [5]. Within luminal-like tumors, we distinguish luminal A and luminal B subtypes, especially luminal A, which is the most common form of breast cancer. Luminal A tumors represent tumors with ER and/or PR positivity, and HER2 negativity (ER+/PR±/HER2−). If the Ki-67 labeling index is determined, the luminal A subtype is defined as a low Ki-67 labeling index (<14%). Luminal B contains three subgroups: Luminal B (HER2 negative) shows ER and/or PR positivity, HER2 negativity, and Ki-67 ≥ 14% (ER+/PR±/HER2−); the luminal B (HER2 positive) type shows ER and/or PR positivity, HER2 positivity, and any Ki-67 (ER+/PR±/HER2+); and the HER2 overexpression type represents HER2 overexpression, with ER and PR absent. Luminal tumor response to hormone treatment is favorable, although they show only slight improvement with traditional chemotherapy [6]. The basal subtype consists of triple-negative (ER−/PR−//HER2−/) tumors, mirroring the expression patterns of basal epithelial cells found elsewhere in the body and normal myoepithelial cells of the breast. Hormone receptors and HER2 may be absent or weakly expressed, while basal markers (keratins 5, 6, 14, 17, EGFR) and proliferation-related genes are abundantly expressed [5]. Triple-negative tumors are notable due to their aggressive clinical trajectory and the absence of established targeted systemic therapy, attributed to the absence of specific receptors. To categorize patients into treatment subtypes that deliver clinical efficacy, it is crucial to perform immunohistochemistry assessments for the presence of ESR, PGR, HER2, and the proliferation marker MKI67 biomarkers. High-throughput gene expression analysis platforms like microarrays have revealed that the response of tumor cells to treatment is determined not by anatomical prognostic factors but by intrinsic molecular attributes that are accessible through molecular techniques [7]. This concept shift introduced a new approach to the stratification and treatment of breast cancer patients. Nevertheless, it is important to note that DNA alterations or shifts in RNA expression do not consistently manifest in protein expression patterns that reflect the biological alterations in cellular function, which are pivotal for targeted therapies and clinical diagnostic tests to be effective [8]. Proposed newer classifications based on protein expression profiling aim to provide more dependable insights into the functional phenotypic differences that underlie the heterogeneity of breast cancer. Asleh et al., with their proteomic study of breast cancer tissue, proved that proteome analysis reveals distinct breast cancer subtypes with differential immune responses and clinical outcomes [9].

B cells are well known for their key role in the development of humoral immune responses, exerting crucial functions in both innate and adaptive immunity. Recently, B cells have drawn attention to breast cancer pathology owing to their impact on tumor regression, prognosis, and treatment response, in addition to their roles in antigen presentation, immunoglobulin production, and regulation of adaptive responses [10]. Recent studies pointed out a significant enrichment of memory B cells in breast cancers compared to healthy tissue, consistently correlating with favorable prognosis in patients with TNBC [11,12]. The presence of tumor-infiltrating lymphocytes (TILs) at diagnosis, comprising both T cells (TIL-Ts) and B cells (TIL-Bs), correlates with better survival following adjuvant systemic therapy and the achievement of pathological complete responses to neoadjuvant chemotherapy in BC [13]. Tumor-infiltrating B cells may exert both pro-tumor and antitumor effects depending on the composition of the tumor microenvironment, the phenotypes of B cells present, and the antibodies that they produce. Tsuda et al. in their study of the B cell population in BC patients and healthy controls observed that the total B cell population was significantly increased in the BC patient group compared to controls, indicating that B cells may have a role to play in tumor promotion [12].

### B Cells: Physiological Differentiation, Maturation, and Function

The initial stages of B cell development start in the bone marrow, where hematopoietic stem cells (HSCs) (CD34+ CD19−) undergo differentiation upon antigen stimulation. This process involves a sequential progression through various developmental stages, differentiating into memory B cells and plasma cells (PCs). Recent single-cell RNA sequencing (scRNA-seq) investigations of breast cancer tissue have even identified up to 13 distinct TIL-B phenotypes, covering the entire spectrum from naïve B cells to plasma cells (PCs) [14].

During the stages of their development, B cells express distinct surface markers, facilitating their immunohistochemical identification. This includes early-stage pro-B cells (CD19+ CD10+ CD34+ IgM−), followed by pre-B cells (CD19+ CD10+ CD34− IgM−), to specified differentiation, presented in Table 1 [15].

Transitional B cells represent an important checkpoint for peripheral tolerance. Research conducted on CD4+ and CD8+ T cells, as well as cell lines, has shown that transcriptome analysis alone seems to be inadequate for predicting protein content; thus, its ability to forecast cellular behavior is limited for a significant portion of genes [16,17]. The absence of quantitative proteomic data on primary B cells presents a barrier to achieving a comprehensive understanding of B cell identity and responsiveness.

## 2. Results

For proteomic analysis of B lymphocytes, we used peripheral blood samples from newly diagnosed breast cancer (BC) patients before any therapy. These patients were IHC characterized into four basic BC categories: luminal A, luminal B, TNBC, and benign. As the control group, the blood of volunteers without the presence of cancer was used. The label-free shotgun proteomic method was employed for protein quantification, using the intensities of identified peptides derived from isolated and digested B lymphocytes. These peptides were analyzed via liquid chromatography and high-resolution mass spectrometry (LC-HRMS/MS), followed by automated database searching. Data were processed via Thermo Proteome Discoverer 2.5.0.400 software. We obtained more than 7408 identified proteins in all groups, 3846 of which were presented in every monitored group and passed the criteria of having at least 2 unique peptides and high confidence identification (Table S1). The coefficient of variation (CV) for the number of identified proteins was 12% in the control group, 25% in luminal A, 34% in luminal B, 36% in benign, and 31% in TNBC groups. To evaluate the differences in the protein expression in the CD19+ lymphocytes, we used label-free quantification based on ion intensity with stringent criteria: a fold change ≥2 of protein abundance in individual BC groups in comparison to the protein abundance in the control group, a false discovery rate (FDR) ≤ 0.05, high confidence, a minimum of two unique peptides, and correction of the probability score using the Benjamini–Hochberg method. In addition, all proteins that met these criteria were verified to be present in each sample. We selected 18 proteins, presented in Table 2 (upregulated in the BC group) and Table 3 (downregulated in the BC group). Only the Battenin protein was upregulated in luminal A, luminal B, and benign patients and downregulated in TNBC patients.

**Figure 1:**
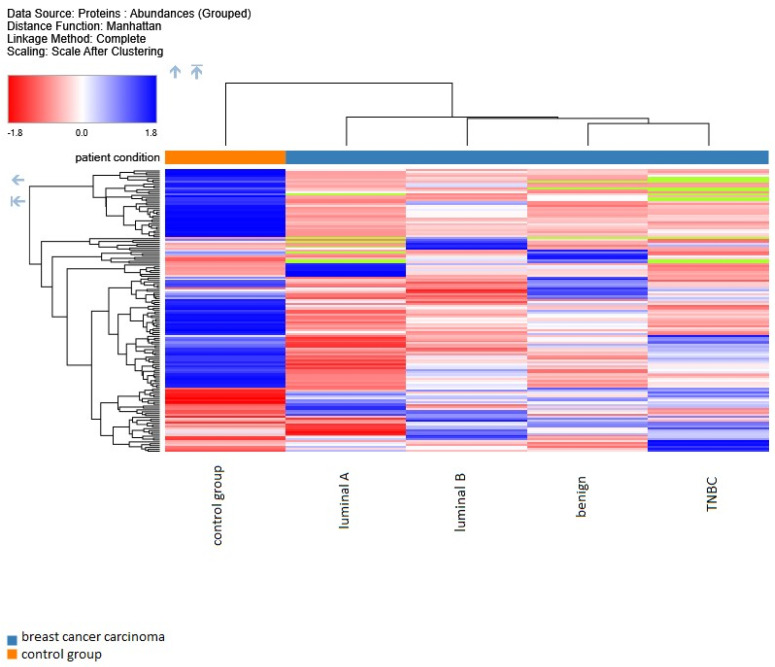
Proteome unsupervised hierarchical clustering reveals distinct breast cancer subtypes versus the control group. Cohort study for the luminal A, luminal B, TNBC, and benign subtypes based on all proteins quantified in every sample and grouped abundances according to the subtype. Data obtained with Proteome Discoverer 2.5.0.400.

Proteomic analysis revealed distinct breast cancer subtypes through hierarchical clustering of proteins in the first stage using the Manhattan distance function applied to measured data, as shown in the heat map in Figure 1. With the proteomic results obtained from B lymphocytes, we can easily divide the patient into the control group and the carcinoma patients (luminal A, luminal B, TNBC, and benign).

**Figure 2:**
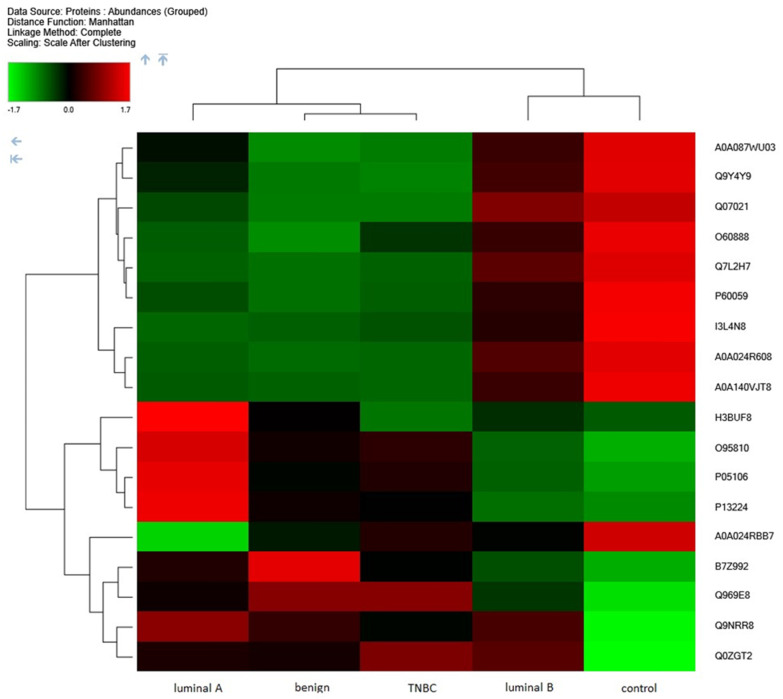
Proteome unsupervised hierarchical clustering applied to 18 selected proteins presented in all groups with an abundance fold change of more than 2, presented in all samples with a high confidence, *p*-value ≤ 0.05. Cohort study for the luminal A, luminal B, TNBC, and benign subtypes, grouped abundances according to the subtype. Data obtained with Proteome Discoverer 2.5.0.400.

After setting criteria to have abundance ratios greater than or equal to 2 or less than or equal to 0.5, with an abundance ratio adjusted *p*-value of less than 0.05 and all proteins identified with high confidence, we obtained 18 proteins. Applying hierarchical clustering to the selected 18 proteins that had significantly changed abundances, we observed a mutual cluster of luminal B and the control group and a second cluster of luminal A, TNBC, and benign patients, as presented in Figure 2.

**Figure 3:**
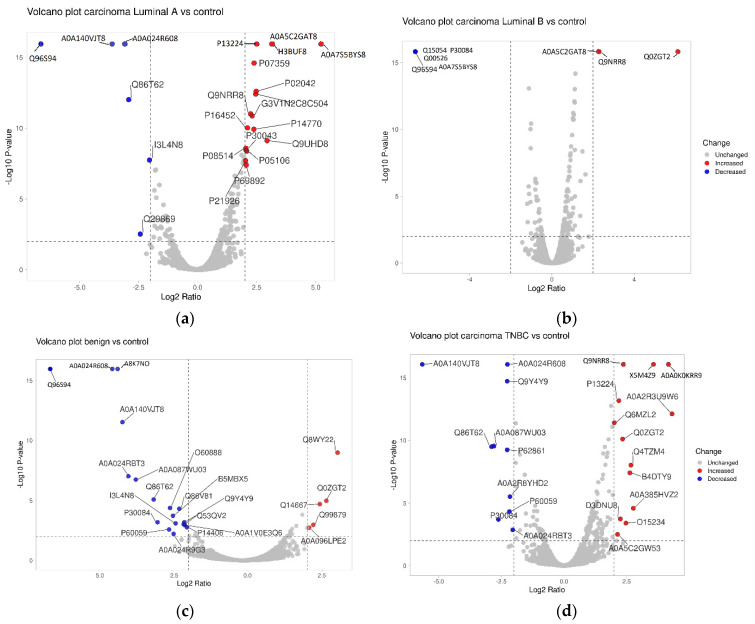
Volcano plot showing significantly changed proteins with log 2-fold change of more than 2, comparing the control group vs. luminal A (**a**), luminal B (**b**), benign (**c**), and TNBC (**d**). Results were adjusted for multiple comparisons using the Benjamini–Hochberg method. The *x*-axis is log2 ratio and the *y*-axis is −log10 (*p*-value). Plot created with the help of https://huygens.science.uva.nl/VolcaNoseR/ (accessed on 14 February 2024).

By applying a minimum log two-fold change to 1.5 between the control group and the luminal B patients, it becomes feasible to differentiate between them as well. The volcano plot, adjusted for multiple comparisons using the Benjamini–Hochberg method, shows significantly changed proteins with a log two-fold change of 2 and a *p*-value ≤ 0.05, comparing the control group versus luminal A, luminal B, benign, and TNBC separately (see Figure 3).

By making a fold change of at least 1.5 between carcinoma samples compared to the control group, the number of proteins with changed abundances increased to 75. Abundance ratios of identified proteins according to carcinoma type compared to the control group with a fold change of at least 2 are presented in Table 4. Upon subjecting all upregulated proteins identified in each group through single hierarchical clustering to Reactome (https://reactome.org/) (accessed on 14 February 2024), a significant association with the hemostasis pathway and platelet activation, signaling, and aggregation was observed, as depicted in Table 5. It presents 20 upregulated pathways in which these proteins are incorporated, most of them showing platelet influencing.

## 3. Discussion

A major advantage of proteomic technologies lies in facilitating the identification and quantification of multiple proteins at once, as well as the ability to measure multiple biomarkers from a single clinical specimen simultaneously. Shotgun proteomics is advantageous over other methods because it enables global and untargeted protein analysis, thereby allowing the characterization of disease-related changes at the protein level and the identification of potentially novel biomarkers.

The estrogen receptor is a well-known cancer biomarker, and classifying breast tumors based on hormone receptor status has been utilized in routine clinical practice for over four decades. ESR1 positivity and PGR positivity are associated with better survival outcomes than negative ESR1/PGR status [18].

Ösz et al. analyzed proteomic data from four independent datasets of breast cancer cases with a correlation of patient survival [1]. They evaluated the association between survival and the expression of 63 proteins and their phosphorylated forms to confirm their prognostic relevance in breast cancer. They revealed a significant association between patient outcomes and the expression of 33 out of 63 proteins. Twelve proteins demonstrated association only with overall survival (OS), while nine proteins were exclusively associated with relapse-free survival (RFS). Additionally, twelve proteins, including PGR, CDH1, BCL2, NDRG1, CTNNB1, APOD, PARP1, RBM3, and four cytokeratins (KRT18, KRT5, KRT6B, and KRT17), were found to be prognostic for both RFS and OS. Among these, three proteins (KRT18, APOD, and CDH1) and four proteins (PGR, CDH1, CTNNB1, and BCL2) were confirmed to be linked with OS and RFS in at least two independent datasets. Excellent overall survival outcomes were connected with elevated expression levels of E-cadherin and the apoptosis regulatory protein BCL2. Additionally, higher BCL2 levels strongly correlated with extended relapse-free survival, alongside the presence of both estrogen and progesterone receptors. Our observations showed, especially in the TNBC group, extreme CDH1 upregulation (5×), although in all groups we monitored elevated values (less than fold change 2). APOD values were lower than in healthy subjects in the luminal A and TBNC groups; however, the benign and luminal B groups showed overexpression. PGRMC1 was upregulated in all carcinoma groups, more in luminal A and TNBC, and vice versa. PGRMC2 was slightly lowered in all cases, but by less than half in benign patients. Apoptosis regulator BCL2 was downregulated in all carcinoma patients, as well as in the most benign and luminal A patients.

Table 2 and Table 3, along with the Battenin protein, show 18 proteins identified across all carcinoma subgroups, each displaying a significant fold change over 2 compared to the proteins in the control group.

We observed strong downregulation in Large ribosomal subunit protein P1 (RPLP1). He et al. in their study detected high levels of RPLP1 expression in TNBC cell lines MDA-MB-231, MDA-MB-436, MDA-MB-468, MDA-MB-453, and tissue samples [19]. They also found that high RPLP1 expression was statistically correlated with poor overall survival. Other research studies have reported that RPLP1 is associated with the progression of colon cancer and gynecologic tumors. He at. al experimentally knocked down RPLP1 expression, and they observed that the epithelial cell marker E-cadherin was increased. Loss of E-cadherin expression is frequently represented in invasive lobular breast carcinoma, which is three times more likely to metastasize [20]. In B lymphocytes E-cadherin was slightly upregulated in benign and luminal A patients, highly upregulated in the luminal B group, and by up to five times in TNBC patients.

Ribonuclease inhibitor (RI), also termed “angiogenin inhibitor”, acts as the inhibitor of ribonucleolytic activity of RNase A and angiogenin. We observed strong downregulation in B lymphocytes in all groups, except carcinoma luminal B (approximately 0.5 downregulated). The expression of RI has been investigated in melanoma and bladder cancer cells. In their study, Tang observed that overexpression of RI induces autophagy in human colorectal cancer, and they speculate that upregulation of RI may represent a novel strategy for the treatment of human CRC [21].

Heterogeneous nuclear ribonucleoprotein D-like (hnRNPDL) was strongly downregulated in all investigated groups, except carcinoma luminal B (only 0.5 downregulated). The hnRNP family comprises 33 proteins characterized by the presence of one or more RNA-binding domains (RBDs) containing amino acid consensus sequences that mediate their interaction with RNA. hnRNP networks are essential for cellular homeostasis, and their dysregulation is associated with cancer and other diseases. Various hnRNP-mediated molecular mechanisms regulate cancer-immune crosstalk as they affect several signaling pathways critical in cancer—hnRNP increases tumor proliferation, migration, and metastasis; metabolic deregulation; and therapeutic resistance [22].

Protein transport protein Sec61 subunit gamma (SEC61G) is strongly downregulated in all groups. SEC61G is a component of the Sec61 complex located in the membrane of the human endoplasmic reticulum. Gao, in his study of hepatocellular carcinoma (HC), revealed a potential relevance in glioblastoma multiforme and observed its high overexpression in HC tissues. SEC61G is required for cell migration and invasion; thus, the role of SEC61G in tumor transfer is important. High expression of SEC61G is relevant to the short survival time of HC patients [23]. Lung adenocarcinoma studies have shown that the (SEC61G) is overexpressed in several tumors and could serve as a potential prognostic marker or target for the diagnosis and treatment of HC [24]. 

Strong downregulation of Protein CutA (CUTA) in B-lymphocytes was observed. It is known as the Acetylcholinesterase-associated protein—the copper-related protein. Since CUTA is predicted to be a mitochondrial protein and is usually connected with Alzheimer’s disease, there are some new observations about its overexpression in the cancer tissue. The expression of the CUTA gene is also upregulated in breast cancer [25].

Actin, cytoplasmic 2 (ACTG1), or γ-actin was strongly downregulated in all patients. ACTG1 is one of seven known actin isoforms in a higher mammal. Its impact on tumor development was highlighted recently by several studies, where its strong expression was associated with an increase in metastatic potential and a worse prognosis for hepatocellular carcinomas and colorectal, lung, and cervical cancers [26]. As bio-informatic research has shown, ACTG1 was highly upregulated in gall bladder cancer and suggested to be used as a biomarker for the early stage of this type of cancer [27]. Downregulation of ACTG1 resulted in the suppression of growth in A431 skin cancer cells, whereas its overexpression significantly promoted cell growth [26]. Abnormal actin isoform expression has been reported in many cancers, so the expression of cytoplasmic actin isoforms hypothetically could be used as a marker for early cancer diagnostics, indicating the efficacy of the currently used treatment.

U6 snRNA-associated Sm-like protein (LSM5) was strongly downregulated. It functions in pre-mRNA splicing, serving as a component of the U4/U6-U5 tri-snRNP complex involved in spliceosome assembly, and as a component of the precatalytic spliceosome (spliceosome B complex). There are two different kinds of spliceosomes in eukaryotic cells: the U2-dependent major spliceosome and the U12-dependent minor spliceosome. While both spliceosomes share structural and functional similarities, each contains five U snRNAs, but the composition of these snRNAs in each spliceosome type is distinct. U6 snRNA is a part of the U2-dependent spliceosome. Aberrant splicing is observed in many types of cancers, so changes in LSM5 expression can point to the presence of cancer changes [28].

Complement component 1, q subcomponent-binding protein mitochondrial (C1QBP)—in all carcinoma groups, the downregulation was significantly decreased. Scully investigated the role of C1QBP as a promising molecular target in breast cancer progression, focusing on its impact on cancer cell growth, especially in TNBC MDA-MB-231 cells. Their results confirm the important role of C1BP in maintaining metabolic activities and showed that its depletion led to changes in cell metabolism and decreased cell proliferation. Experimental silencing of C1QBP in MDA-MB-231 cells led to decreased cell proliferation and a slower rate of cell growth. Similar observations were noted in prostate cancer cells as well [29].

Nucleosome assembly protein 1-like 1 (NAP1L1) was downregulated in all carcinoma groups, especially carcinoma luminal A. NAP1L1 is suggested to have an oncogenic role in several tumors based on its overexpression [30,31]. Suppression of NAP1L1 expression by small interfering RNA (siRNA) or small hairpin RNA (shRNA) methods significantly decreased cell proliferation in vivo and in vitro [31].

Eukaryotic translation initiation factor 3 subunit M (EIF3M) was strongly downregulated in all groups, with only carcinoma luminal B to 0.734. The EIF3 gene family is essential in controlling translation initiation during the cell cycle. Song et al. examined the transcriptional expression levels, clinical prognostic significance, and survival value of individual EIF3 subunits in breast cancer by performing comprehensive bioinformatics analysis on several large online databases with a combination of experimental verification, and they confirmed the crucial function of EIF3 in breast cancer. EIF3M was highly expressed in breast cancer tissues. They suggested EIF3 subunits to be novel diagnostic and prognostic markers and therapeutic targets for breast cancer [32].

Integrin β3 (ITGB3) was upregulated in all groups, especially carcinoma luminal A and TNBC patients. ITGB3 likely contributes to skeletal metastasis through its modulation of gene expression, a common complication in the progression of breast cancer. The levels of ITGB3 were examined in exosomes from the rat plasma with skeletal metastases and from MDA-MB-231 cells incubated with these vesicles. ITGB3 has emerged as a promising therapeutic target for breast cancer skeletal metastasis. Various approaches to inhibit ITGB3 were explored, yielding promising results [33].

Caveolae-associated protein 2 (CAVIN2) was upregulated in all groups at least two times. Cell membranes contain small invaginations called caveolae. The formation of caveolae relies on two protein classes: caveolins and cavins, which form extensive complexes enabling self-assembly into caveolae structures. In cancers, the expression of caveolins and cavins varies, undergoing alterations both within tumors and in the surrounding stromal cells of the tumor microenvironment. Significantly, their expression and function have been linked to resistance against numerous cancer drugs [34].

Pre-rRNA-processing protein (TSR2) was upregulated more than twice in all groups, with only luminal B showing 1,6 times. According to the last observations, TSR2 releases small ribosomal subunit protein eS26 (RPS26) from mature ribosomes to remodel ribosome populations. Moreover, TSR2 stores free RPS26 and promotes re-incorporation of the protein, thereby repairing the subunit after the stress subsides. Moreover, insufficiency of ribosomal proteins can lead to the accumulation of ribosomes lacking these proteins and, in human cells, predispose them to cancer [35].

Battenin (CLN3) is a transmembrane protein implicated in numerous biological functions, such as osmoregulation, autophagy, apoptosis, lysosomal pH regulation, ceramide metabolism, cell cycle modulation, cell migration, and proliferation. It has been verified to exhibit increased expression levels in prostate cancer, ovarian cancer, colon cancer, breast cancer, neuroblastoma, glioblastoma, and hepatocellular carcinoma. When CLN3 was suppressed, carcinoma cell lines experienced a notable decrease, which was also confirmed in human tumor xenografts in mice. The expression and function of CLN3 may serve as predictive markers of patient prognoses in the mentioned cancers and also serve as therapeutic targets [36]. In our observation, this protein passed all criteria but was strongly upregulated in the luminal A group, with a nearly twofold increase in expression in luminal B and benign groups. Conversely, it exhibited significant downregulation in TNBC, with a threefold decrease.

CDC42 small effector protein was strongly upregulated in all cases. It is a member of the Rho family of small GTPases and a master regulator of the actin cytoskeleton, controlling cell motility, polarity, and cell cycle progression. Recent studies show that in most human cancers CDC42 is abnormally expressed and promotes neoplastic growth and metastasis. In her work, Murphy concluded that CDC42 and its regulatory and effector protein partners continue to demonstrate an ever-central role in the molecular subversion of signaling in cancer [37].

Gelsolin (GSN) was upregulated in all groups, especially the benign group. Its extracellular isoform ranks belong to the most abundant circulating plasma proteins and can be found as a novel diagnostic biomarker for early disease detection. Current evidence indicates that gelsolin can serve as either an oncoprotein or a tumor suppressor, contingent upon the specific carcinoma type. Notably, it exhibits high expression levels in pancreatic ductal adenocarcinoma cells and substantially influences cancer motility. Furthermore, chemo-resistant gynecological cancer cells demonstrate elevated levels of gelsolin expression, and its overexpression correlates with the aggressive behavior exhibited by gynecological cancer cells. Observations indicate a continuous increase in plasma GSN levels throughout the progression of cervical carcinoma. Experimental knockdown of the GSN gene in cervical carcinoma cells resulted in reduced activation of matrix metalloproteinase-2 (MMP-2), suggesting that gelsolin regulates the epithelial–mesenchymal transition (EMT) activity of cervical cancer cells by enhancing proteolysis to facilitate tumor invasion. To date, several clinical trials have been conducted to examine the diagnostic and therapeutic potential of plasma GSN by using neutralizing antibodies [38].

We observed exceptionally high upregulation of nexilin (NEXN) in all cancer groups, with a nearly 70-fold change in luminal B patients, and the least upregulated was up to 5-fold in TNBC patients. Nexilin plays a critical role in actin cytoskeletal remodeling, and according to recent research is involved in cancer metastasis [39]. Dang focused on B-cell receptor-associated protein 31 (BAP31), which is highly expressed in primary hepatocellular carcinoma, cervical cancer tissue, and ovarian, breast, liver, esophageal, rectal, and lung cancers, indicating it is a potential carcinoma biomarker and has a potential role in future anticancer therapy. Our measurements showed that BAP31 concentration in B lymphocytes was slightly increased in carcinoma patients, while it was downregulated in benign patients. Depletion of BAP31 expression in Dang’s experiment on HeLa cells resulted in the decreased expression of Nexilin, together with Drebrin, M-RIP, and SPECC1L, with a uniform distribution in the cells, indicating that BAP31 could regulate the expression and subcellular localization of these four metastasis-related proteins.

Platelet glycoprotein Ib beta chain (GP1BB), upregulated in all cases, is presented on platelets and functions as a receptor for the von Willebrand factor as an important factor in platelet adhesion to wound sites to form platelet plugs. It forms a heterodimer with GP1B beta subunits and associates with GP-IX, facilitating platelet adhesion and initiating thrombus formation. We observed the von Willebrand factor to be significantly upregulated in all cases, and the most in the luminal A group. Table 5 presents 20 upregulated pathways in which proteins connected with platelet influencing are incorporated: platelet activation, signaling, and aggregation; platelet degranulation; response to elevated platelet cytosolic Ca2+; defective F9 activation (the F9 gene provides instructions for making a protein called coagulation factor IX); defects of platelet adhesion to exposed collagen; GP1b-IX-V activation signaling (involved in platelet clearance, platelet formation, and thrombopoietin generation, and thus is a key molecule controlling the platelet equilibrium); platelet adhesion to exposed collagen; defects of contact activation system and kallikrein/kinin system; diseases of hemostasis; intrinsic pathway of fibrin clot formation; formation of fibrin clot (clotting cascade); and platelet aggregation. We also observed upregulation of scavenging of heme from the plasma pathway. Scavenger receptors belong to a superfamily of proteins that are structurally heterogeneous and encompass the miscellaneous group of transmembrane proteins and the soluble secretory extracellular domain and are classified based on their nucleotide sequence alignment and protein structure. They are functionally diverse, as they are involved in various disorders and immunity-related signaling pathways, and their major function is innate immunity and homeostasis. Further research is required to investigate and confirm the potential role of scavenger receptors in leukocyte migration to other tissues such as the spleen and lymph nodes [40].

Previous reports showed that about 20% of cancers are correlated with chronic inflammation caused by infections, exposure to irritants, or autoimmune disease. On the other hand, oncogenic changes are characterized by inflammatory tumor microenvironment in the premalignant and malignant cells. Thus, there is a cross-interaction between inflammation and cancer [41]. Platelets are critical for hemostasis and thrombosis, but recent research highlights their role in many other processes, including inflammation, wound healing, and lymph angiogenesis.

Platelets as small anucleate cells are traditionally described as the major effectors of hemostasis and thrombosis. They have been recognized as an immunoregulatory cellular component [42]. Upon activation, platelets release cytokines, chemokines, growth factors, and platelet-derived microparticles (PMPs) and express a set of activation molecules on their membrane (P-selectin and CD40L) that allow the platelet to bind to leukocytes. Platelets directly modulate humoral activity, stimulating B cell proliferation, antibody production, and the membrane expression of CD27 and CD86, via soluble CD40L. Platelet-derived soluble factors (TGFβ, PF4, and CD40L) and the binding of platelets to leukocytes (CD62P-PSGL1) decrease T cell proliferation and inflammatory cytokine production and increase IL-10 production by T lymphocytes and monocytes [43]. In his study, Zamora confirmed that levels of lymphocytes with bound platelets were higher in systematic lupus erythematosus patients than in healthy donors. In their in vitro study, Oleksowicz demonstrated in 1997 that the ability of tumor cells to aggregate platelets correlates with the tumor’s metastatic potential, and inhibition of tumor-induced platelet aggregation has been shown to suppress metastases in rodent models [44]. However, increasing evidence indicates that platelets play several roles in the progression of malignancies and cancer-associated thrombosis. Platelets are suspected to provide a shield for tumor cells against the immune system. This hypothesis was substantiated by a study revealing that platelets aid in the spread of metastasis protecting tumor cells from natural killer (NK) cell lysis. The proposed mechanism may depend on platelets’ capacity to excrete substances like transforming growth factor-β (TGF-β), which can suppress the expression of natural killer group 2 member D (NKG2D) on NK cells, thereby diminishing their cytotoxic impact [45]. In all groups, we observed nearly twice the upregulation of TGFB, but TNBC was the highest. Recent research suggests a reciprocal interaction between platelets and tumors, facilitated by RNA transfer and extracellular vesicles. Additionally, there is growing interest in the potential therapeutic application of antiplatelet agents in cancer treatment [46]. It appears that cancer cells can alter the physiology and phenotype of platelets, affecting their RNA profiles, circulating numbers, and activation states. Certainly, platelet activation triggers the release of active biomolecules, including platelet-derived microparticles and the contents of their granules, all of which contribute to tumor progression and the facilitation of successful metastatic dissemination [47]. The last discoveries confirm that after platelet activation, although they are anucleated, protein synthesis is also possible [48]. Yari’s study investigates the impact of platelet-derived microparticles (Plt-MPs) on an immortalized B-cell line, which serves as a surrogate for peripheral blood B cells. He demonstrated that Plt-MPs can trigger the activation and differentiation of immortalized B-cell origin cells [49].

By label-free shotgun proteomic analysis, 18 significantly altered proteins were identified, which are potential candidate markers for various types of cancer according to recent studies. Of course, more detailed studies on a larger group of patients are needed to confirm their potential also as breast cancer markers. We observed the correlation between the number of these proteins identified in carcinoma cells and B cells, and we revealed that their presence is in opposite amounts to each other. When the monitored protein is, according to the latest studies, upregulated in cancer cells, we notice its downregulation in B lymphocytes and vice versa.

Guo’s study of MK-2206 drug treatment induced the most significant differences between the TNBC and non-TNBC cell lines at the proteome level [50], confirming the great potential of proteomic research. We identified 129 significantly upregulated proteins and 86 downregulated proteins in B lymphocytes in TBNC patients in comparison to the control group.

## 4. Materials and Methods

### 4.1. Patient Selection

This study was approved by the Human Research Ethics Committee of Louis Pasteur University Hospital in Košice. The informed consent (2020/EK/06407) was obtained in written form from women in the control group, as well as the breast cancer group (comprising benign, luminal A, luminal B, and TNBC groups). For the proteomic analysis, peripheral blood samples from newly diagnosed breast cancer (BC) patients before any therapy were used. The randomized peripheral blood samples from the control group were obtained from the Department of Gynecology and Obstetrics, while the samples from the BC groups were taken from the 1st Department of Surgery of the Faculty of Medicine, Pavol Jozef Šafárik University in Košice and the L. Pasteur University Hospital in Košice. Control samples comprised 20 patients without the presence of cancer with a mean age of 68 years (in the range of 39–85 years). Carcinoma groups consist of 8 samples from benign patients, with a mean age of 53 years (between 39–73 years); 8 patients with confirmed luminal A carcinoma, with a mean age of 69 years (between 57–76 years); 8 samples from patients with confirmed luminal B breast cancer, with a mean age of 61 years (ranging from 46 to 81 years); and 3 samples were taken from patients with TNBC, with a mean age of 72 years (from 57 to 83 years). Patient selection according to the IHC result was carried out by the accredited medical laboratory, registration number 158/Q-044, certified according to ISO 9001:2025, and certificate valid until April 2026. Accreditation was completed by SNAS (Slovak National Accreditation Service), which is a signatory of EA MLA and ILAC MRA in the field of the certification of medical laboratories. Molecular classification of BC using immunohistochemistry defines specific subtypes, such as luminal A (ER+, PR+, HER2−, Ki67 < 14%), luminal B (ER+, PR+, HER2−, Ki67 ≥ 14%), and TNBC (ER−, PR−, HER2−, high Ki67) [6].

### 4.2. Blood Sampling and B Cell Isolation

Peripheral blood samples of 10 mL volume each were collected in BD Vacutainer K2EDTA tubes, cooled to 4 °C, and processed within 30 min from blood sampling. Ten mL of peripheral blood was diluted with 20 mL of saline solution (PBS containing BSA, and EDTA, pH = 7.4) and subsequently centrifuged at 600× *g* for 10 min at 4 °C. The plasma was discarded and filled up to 10 mL with isolation solution. A total of 250 μL of CD19 magnetic beads (Dynabeads™, Invitrogen by Thermo Fisher Scientific, Waltham, MA, USA) washed with isolation solution were added to the diluted blood and mixed in a multi-rotator (Multi Bio RS-24, Biosan, Riga, Latvia) at 4 °C for 20 min. A test tube with the sample and magnetic beads was inserted into a magnetic stand (DynaMag™-50 Magnet, Invitrogen by Thermo Fisher Scientific). The captured magnetic beads were washed 3 times with the isolation solution and finally transferred to 50 mM ammonium bicarbonate buffer (ABC, AppliChem GmbH, Darmstadt, Germany). Samples were sonicated for 1 h at 4 °C and released magnetic beads were dismissed with the help of a magnetic stand. In total, 200 µL of isolated supernatant were precipitated with 1800 µL of cold ethanol at −20 °C during the night. Precipitated proteins were dissolved in 150 µL of ABC solution. Protein concentration was determined using the BCA method with a QuantiPro BCA Assay Kit (Sigma-Aldrich, St. Louis, MO, USA). This measurement was then used to calculate the required amounts of additional reactants needed for protein digestion. The cysteine disulfide bonds were reduced with 0.025 M dithiothreitol solution (purity ≥ 98%, Bio-Rad, Hercules, CA, USA) and the samples were then incubated in a thermomixer at 60 °C for 30 min. Subsequently, alkylation with 0.25 M iodoacetamide solution (purity ≥ 99%, Bio-Rad) in the dark at 21 °C was performed for 30 min. The samples were diluted 2 times with 5 mM CaCl_2_ (purity ≥ 98%, Merck, Darmstadt, Germany) and digested with trypsin (Roche, Basel, Switzerland) in a ratio of trypsin/sample = 1:40 (*w*/*w*) overnight at 21 °C. Digestion was stopped by adding 20% formic acid (Merck) to obtain pH = 3. The sample volume was adjusted using the rotary evaporator SpeedVac (Labconco, Kansas City, MO, USA) to a concentration of 1 µg·mL^−1^. Samples were centrifuged at 18,000× *g*, 4 °C, and the supernatant was diluted with loading mobile phase, water: acetonitrile (2/98, *v*/*v*) containing 0.1% HCOOH, to a final concentration of 0.04 µg·mL^−1^ before injection to LC-MS/MS.

### 4.3. LC-MS/MS Analysis and Database Search

Samples were analyzed by LC-MS/MS using a Vanquish™ Neo UHPLC System coupled to a high-resolution, accurate-mass (HRAM) Orbitrap Exploris™ 480 Mass Spectrometer (Thermo Fisher Scientific). Peptides isolated from B lymphocytes with an injected volume of 10 µL were trapped at PepMap™ Neo Trap Cartridge C 18 (5 mm, 5μm particle) with a flow of 60 μL·min^−1^, followed by separation on a Thermo Scientific™ EASY-Spray™ analytical column (500 mm, 2 μm particle) at a temperature of 40 °C. The composition of mobile phase A was 0.1% HCOOH in water, and the composition of B was 0.1% HCOOH in a water: acetonitrile (20/80, *v*/*v*) mixture as follows: in a 100 min linear gradient change 2–24% solvent B, for the next 20 min up 40% B, for the next 10 min up to 90% B, and held for 10 min at a flow rate of 300 nL·min^−1^. The mass spectrometer was operated in positive data-dependent acquisition mode (cycle time). MS1 spectra were measured with a resolution of 120,000 and a maximum injection time of 100 ms. The scan range was 350–1700 Da, the MS/MS scan resolution was 30,000, and the maximum injection time was 50 ms, with an HCD fragmentation type with 30% collision energy and an isolation window of *m*/*z* = 2. The dynamic exclusion was set to 15 s with a 10 ppm mass tolerance around the precursor and its isotopes. Before each injection, an automatically performed internal mass check (one-point-mass calibration) for fluoranthene was used.

**Figure 4:**
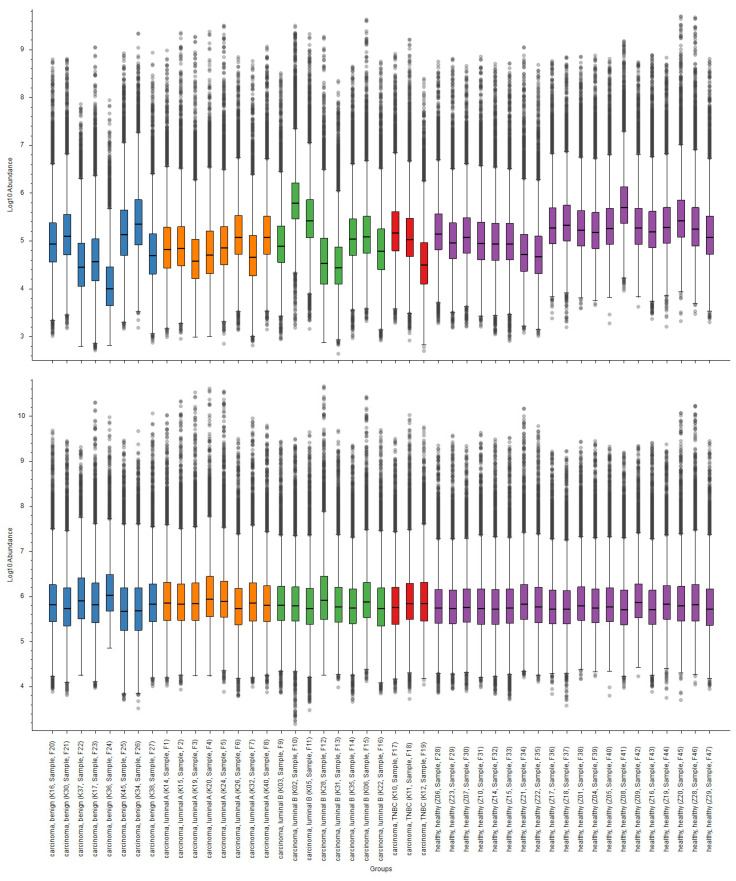
Distribution of protein abundances before and after normalization, identified in samples, with blue—benign, orange—luminal A, green—luminal B, red—TNBC, and violet—control group, using Proteome Discoverer version 2.5.0.400 Thermo Scientific™ software, specific protein amount normalization.

The acquired raw run files were analyzed using the Proteome Discoverer (PD) version 2.5.0.400 (Thermo Scientific™, Waltham, MA, USA) software. Protein identification was performed using the combination of three search engines available in PD, MS Amanda 2.0, SequestHT, and Mascot utilizing the Uniprot Homo sapiens database (release 2023_02, which contained 182,026 sequences) for MS Amanda, and SequestHT and Swiss-Prot Homo Sapiens database for Mascot. Proteolytic enzyme specification during the search was trypsin, cleaving after lysine (K) and arginine (R) except when followed by proline (P). The fixed modification was set as carbamidomethylation of cysteine, and variable modifications were set as oxidation of methionine, N-terminal acetylation, N-terminal methionine lost, and N-terminal methionine lost + acetylation. Mass tolerances for precursor and fragment ions were set at 10 ppm and 0.02 Da, respectively. The minimum cutoff for peptide length was set at six amino acids, and the maximum permissible missed cleavage was set at two. The identified spectra were rescored using a percolator. For Label-Free Quantification (LFQ), both unique and razor peptides were used, with precursor abundance in the samples compared based on the integration of the identified peptide intensities. Protein groups were considered for peptide uniqueness, and shared quantitative results were used. The statistical significance of the LFQ results was determined using a background-based *t*-test with a predefined summed abundances setting, where protein abundances were calculated by summing the sample abundances of the connected peptide groups. Pairwise ratio-based analysis was used for protein ratio calculation. We applied a normalization step with a specific protein amount using a scaling mode on all averages to eliminate systematic technical variations from sample preparation and instrument performance, which was the most suitable normalization method available directly in the PD software. For normalization, all peptides were used. The results after normalization are presented in Figure 4. We applied a non-nested design with no imputation method; extreme ratio values (100; 0.01) were not considered for median selection.

## 5. Conclusions

A liquid biopsy is a simple and less invasive alternative to surgical biopsies that enables doctors to discover a range of information about a tumor through a simple blood sample. Traces of cancer DNA as well as “marker” protein dysregulation in the blood can give information about cancer’s presence and then which treatments will be most suitable for that patient.

According to our preliminary results, a significant correlation was observed between patient treatment selection based on immunohistochemistry with a comparison of those detected by proteomic technologies, where cluster analysis safely distinguishes carcinoma samples from control ones. The 18 characterized proteins that were significantly changed in all groups are candidates to serve as breast cancer indicators in blood, after other monitoring of the larger patient group. With the method specified to extract CD-19 cells, we identified proteins that confirmed that platelets interacting with B-lymphocytes and activated platelet pathways can indicate a probable cancer presence.

## Figures and Tables

**Table 1:** B cell cluster of differentiation (CD markers).

B Cell Subtype	Immunohistochemical Markers
Naïve B cell	CD19+, CD20+, IgM+IgD−, CD38−/−
Naïve activated B cell	CD19+, CD20+, IgM+, IgD+, CD38+
Germinal center B cell	CD19+, CD20+, IgM+/−, IgD+, CD38++
Plasmablast	CD19+, CD20−, CD38++, CD27++, IgD−, IgM/G/A/E+
Plasma cell	CD19+/−, CD20−, CD38++, CD138+, CD27+ IgD−, IgM/G/A/E+
Memory B cell	CD19+ CD20+ CD38−, CD27+, IgD−, IgM/G/A/E+

**Table 2:** Upregulated proteins presented in luminal A, luminal B, TNBC, and benign.

Accession	Description	Coverage [%]	Peptides	Unique Peptides	MW [kDa]
B7Z992	Gelsolin	61	37	2	78.8
P13224	Platelet glycoprotein Ib beta chain	25	6	6	21.7
P05106	Integrin beta-3	58	40	4	87
Q9NRR8	CDC42 small effector protein	24	2	2	8.9
O95810	Caveolae-associated protein 2	54	21	21	47.1
Q969E8	Pre-rRNA-processing protein TSR2 homolog	19	5	5	20.9
Q0ZGT2	Nexilin	12	7	7	80.6

**Table 3:** Downregulated proteins presented in luminal A, luminal B, TNBC, and benign.

Accession	Description	Coverage [%]	Peptides	Unique Peptides	MW [kDa]
A0A024R608	Ribosomal protein, large, P1, isoform CRA_a	85	7	6	11.5
A0A024RBB7	Nucleosome assembly protein 1-like 1, isoform CRA_a	56	18	16	45.3
A0A087WU03	Heterogeneous nuclear ribonucleoprotein D-like	40	2	2	6.7
A0A140VJT8	Ribonuclease inhibitor	88	34	2	49.9
I3L4N8	Actin, cytoplasmic 2	99	49	4	39
O60888	Protein CutA	49	5	5	19.1
P60059	Protein transport protein Sec61 subunit gamma	29	2	2	7.7
Q07021	Complement component 1 Q subcomponent-binding protein, mitochondrial	54	10	10	31.3
Q7L2H7	Eukaryotic translation initiation factor 3 subunit M	51	15	15	42.5
Q9Y4Y9	U6 snRNA-associated Sm-like protein LSm5	56	6	6	9.9

**Table 4:** A total of 17 down- and upregulated proteins identified in luminal A, luminal B, TNBC, and benign samples with an abundance ratio change (AR) of more than 1.5 to the control group, with coefficient of variation (CV) of protein abundances within each group.

Description	Gene Symbol	Accession	AR Benign/ Control	CV [%] Benign	AR Lumi-nal A Control	CV [%] Lumi-nal A	AR Lumi-nal B/Control	CV [%] Lumi-nal B	AR TNBC/Control	CV [%] TNBC
**DOWN REGULATED**										
*Ribosomal protein, large, P1, isoform CRA*	RPLP1	A0A024R608	0.043	233.65	0.118	162.09	0.461	86.08	0.208	153.7
*Ribonuclease inhibitor*		A0A140VJT8	0.054	196.44	0.082	128.71	0.456	60.23	0.02	n/a
*Heterogeneous nuclear ribonucleoprotein D-like*	HNRNPDL	A0A087WU03	0.074	223.7	0.347	43.3	0.581	94.49	0.143	108.33
*Protein transport protein Sec61 subunit gamma*	SEC61G	P60059	0.161	147.94	0.287	48.63	0.567	60.72	0.221	50.32
*Protein CutA*	CUTA	O60888	0.164	229.51	0.282	80.01	0.614	76.12	0.511	87.57
*Actin, cytoplasmic 2*	ACTG1	I3L4N8	0.187	157.77	0.243	77.41	0.571	83.21	0.262	88.8
*U6 snRNA-associated Sm-like protein LSm5*	LSM5	Q9Y4Y9	0.228	207.85	0.395	71.81	0.498	78.14	0.208	117.05
*Complement component 1 Q subcomponent-binding protein, mitochondrial*	C1QBP	Q07021	0.296	168.91	0.412	84.96	0.699	41.6	0.51	89.27
*Nucleosome assembly protein 1-like 1, isoform CRA*	NAP1L1	A0A024RBB7	0.32	69.93	0.399	80.34	0.708	59.95	0.511	93.97
*Eukaryotic translation initiation factor 3 subunit M*	EIF3M	Q7L2H7	0.351	171.11	0.367	61.06	0.734	50.91	0.456	84.82
**UP REGULATED**										
*Integrin beta-3*	ITGB3	P05106	2.224	94.2	4.228	97.92	1.583	91.35	3.679	117.97
*Caveolae-associated protein 2*	CAVIN2	O95810	2.262	80.52	2.785	122.56	1.535	48.04	2.583	97.02
*Pre-rRNA-processing protein TSR2 homolog*	TSR2	Q969E8	2.385	30.62	2.1	19.4	1.649	16.28	2.323	29.61
*Platelet glycoprotein Ib beta chain*	GP1BB	P13224	2.428	101.72	5.664	97.69	2.053	115.73	4.556	127.94
*CDC42 small effector protein 1*	CDC42SE1	Q9NRR8	2.519	44.69	4.718	24.56	4.898	34.17	5.186	21.57
*Gelsolin*		B7Z992	3.568	83.17	2.911	77.88	1.779	42.36	2.488	65.93
*Nexilin*	NEXN	Q0ZGT2	6.257	46.96	9.158	49.51	69.689	24.14	5.048	19.48

**Table 5:** Pathways hit by 17 significantly changed proteins presented in all monitored groups—luminal A, luminal B, and TNBC.

Pathway Identifier	Pathway Name	#Entities Found	#Entities Total
R-HSA-76002	Platelet activation, signaling, and aggregation	7	265
R-HSA-114608	Platelet degranulation	5	128
R-HSA-76005	Response to elevated platelet cytosolic Ca2+	5	133
R-HSA-9673221	Defective F9 activation	2	6
R-HSA-9846298	Defective binding of VWF variant to GPIb:IX:V	2	7
R-HSA-9845620	Enhanced binding of GP1BA variant to VWF multimer:collagen	2	7
R-HSA-381426	Regulation of Insulin-like Growth Factor (IGF) transport and uptake by Insulin-like Growth Factor Binding Proteins (IGFBPs)	4	124
R-HSA-9823587	Defects of platelet adhesion to exposed collagen	2	8
R-HSA-2173782	Binding and uptake of ligands by scavenger receptors	4	129
R-HSA-9668250	Defective factor IX causes hemophilia B	2	9
R-HSA-430116	GP1b-IX-V activation signaling	2	12
R-HSA-109582	Hemostasis	7	727
R-HSA-75892	Platelet adhesion to exposed collagen	2	16
R-HSA-9651496	Defects of Contact Activation System (CAS) and Kallikrein/Kinin System (KKS)	2	16
R-HSA-9671793	Diseases of hemostasis	2	19
R-HSA-140837	Intrinsic pathway of fibrin clot formation	2	23
R-HSA-2168880	Scavenging of heme from plasma	3	99
R-HSA-8957275	Post-translational protein phosphorylation	3	107
R-HSA-140877	Formation of fibrin clot (clotting cascade)	2	39

## Data Availability

The data presented in this study are available on the PRIDE platform, project accession: PXD052185.

## Supplementary Materials

The following supporting information can be downloaded at: https://www.mdpi.com/article/10.3390/ijms25137351/s1.
